# Effects of Aβ-derived peptide fragments on fibrillogenesis of Aβ

**DOI:** 10.1038/s41598-021-98644-y

**Published:** 2021-09-28

**Authors:** Faisal Abedin, Nabin Kandel, Suren A. Tatulian

**Affiliations:** 1 Physics Graduate Program, University of Central Florida, Orlando, FL USA; 2 Department of Physics, College of Sciences, Burnett School of Biomedical Sciences, College of Medicine, University of Central Florida, Orlando, FL USA; 3 Present Address: Center for Biotechnology and Interdisciplinary Studies, Rensselaer Polytechnic Institute, Troy, NY USA

**Keywords:** Biophysics, Molecular conformation

## Abstract

Amyloid β (Aβ) peptide aggregation plays a central role in Alzheimer’s disease (AD) etiology. AD drug candidates have included small molecules or peptides directed towards inhibition of Aβ fibrillogenesis. Although some Aβ-derived peptide fragments suppress Aβ fibril growth, comprehensive analysis of inhibitory potencies of peptide fragments along the whole Aβ sequence has not been reported. The aim of this work is (a) to identify the region(s) of Aβ with highest propensities for aggregation and (b) to use those fragments to inhibit Aβ fibrillogenesis. Structural and aggregation properties of the parent Aβ_1–42_ peptide and seven overlapping peptide fragments have been studied, i.e. Aβ_1–10_ (P1), Aβ_6–15_ (P2), Aβ_11–20_ (P3), Aβ_16–25_ (P4), Aβ_21–30_ (P5), Aβ_26–36_ (P6), and Aβ_31–42_ (P7). Structural transitions of the peptides in aqueous buffer have been monitored by circular dichroism and Fourier transform infrared spectroscopy. Aggregation and fibrillogenesis were analyzed by light scattering and thioflavin-T fluorescence. The mode of peptide-peptide interactions was characterized by fluorescence resonance energy transfer. Three peptide fragments, P3, P6, and P7, exhibited exceptionally high propensity for β-sheet formation and aggregation. Remarkably, only P3 and P6 exerted strong inhibitory effect on the aggregation of Aβ_1–42_, whereas P7 and P2 displayed moderate inhibitory potency. It is proposed that P3 and P6 intercalate between Aβ_1–42_ molecules and thereby inhibit Aβ_1–42_ aggregation. These findings may facilitate therapeutic strategies of inhibition of Aβ fibrillogenesis by Aβ-derived peptides.

## Introduction

Extracellular fibrillar deposits of the amyloid β (Aβ) peptide in cerebral parenchyma and vasculature constitute a major histopathological trait of Alzheimer’s disease (AD)^1–3^. The presence of Aβ plaques in brains of AD patients led to the amyloid cascade hypothesis, positing direct involvement of Aβ aggregates in AD etiology^1,4^. The cytotoxicity of Aβ has been documented in numerous in vivo and in vitro studies, lending support for the central role of Aβ in AD^1,3,5–8^.

Aβ is derived from the amyloid precursor protein (APP) through cleavage by β- and γ-secretases^9,10^. Due to poor sequence specificity of these enzymes, Aβ species of varying numbers of amino acid residues are present in human brain. Along with the most prevalent forms, i.e. 42- and 40-residue species (Aβ_1–42_ and Aβ_1–40_), many shorter peptides, generated by post-cleavage enzymatic processing, are also present. In addition, extensive somatic gene recombination in human neurons results in numerous *APP* variants with insertions, deletions, or missense mutations, including early termination codons and mutations linked to early onset AD^11^. In effect, the total Aβ pool in human brain is a heterogenous assembly of peptide species of different size and with distinct structural features and toxicities^12–17^.

**Figure 1:**
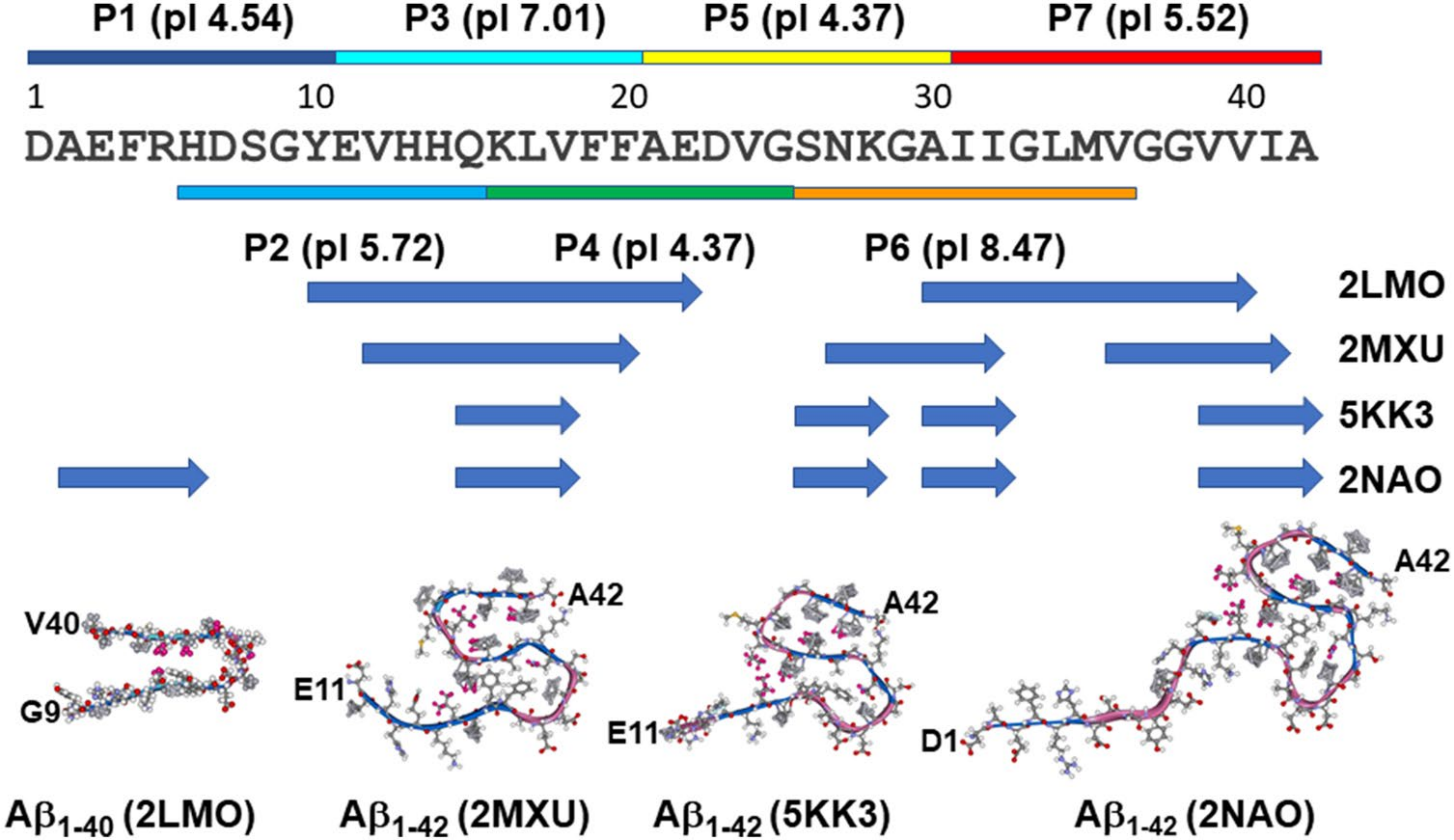
Aβ_1–42_ amino acid sequence in single-letter format, with stretches corresponding to peptides P1 through P7 shown by horizontal bars of different colors above or below the sequence, along with respective theoretical isoelectric points (according to ExPASy ProtParam function, https://web.expasy.org/protparam). Horizontal blue arrows below the sequence show the segments corresponding to β-sheet structures in Aβ_1–40_ or Aβ_1–42_ fibrils determined experimentally, with Protein Data Bank entries shown on the right-hand side. Segments left blank are described as loop or turn regions. In the lower panel, one structure for Aβ_1–40_ and three structures for Aβ_1–42_ in fibrillar form are shown (fibril axis and the direction of interstrand H-bonding are perpendicular to the plane of the image). Backbone regions in β-sheet and turn/loop conformations are colored blue and rose, respectively.

Aβ peptides are inherently polymorphic and undergo dynamic structural transitions during fibrillogenesis^18–29^. Monomers and oligomers can adopt a variety of secondary/tertiary structures, including fractions of α-helix, β-sheet, various types of turn and loop conformations^18–21,30^. Fibrils of Aβ_1–40_ and Aβ_1–42_ acquire characteristic parallel, in-register cross-β structure, i.e. intermolecular β-sheets with H-bonding along the fibrillar axis^24,31–34^. The atomic resolution structures of Aβ_1–40_ and Aβ_1–42_ fibrils have been determined by solid state NMR and other techniques and revealed relatively similar secondary structures but distinct 3-dimensional folds. Aβ_1–40_ monomers in the fibrillar structure adopt a U-shaped conformation with two β-strands (residues 10–22 and 30–40) separated by a loop and with unordered N-terminus^31^ (Fig. 1). In contrast, Aβ_1–42_ forms S-shaped structure with a larger number (3–5) of shorter β-strands^32–34^ (Fig. 1).

Toxicity depends on particular Aβ species, the aggregation state, and the structural features of peptide assemblies^3,20,35–38^. Monomers and mature fibrils are essentially nontoxic or mildly toxic; the main cytotoxic effect is exerted by intermediate oligomeric assemblies^3,20,37–43^. In human brain, Aβ_1–40_ is found in higher concentrations than Aβ_1–42_, but the latter is more toxic, which is believed to result from its increased hydrophobicity and tendency towards β-sheet formation and aggregation^26,35,36,44–48^. Enzymatically modified forms of Aβ are produced in vivo by N-terminal trimming followed by cyclization of glutamic acid, which possess superior cytotoxicity deemed to stem from their distinct aggregation and structural properties such as faster aggregation into β-sheet fibrillar assemblies^49,50^ or stabilization of toxic oligomeric structures^5^. Apparently, the aggregation pathways and accompanying structural transitions in various Aβ species underlie the ultimate cytotoxic effects. Therefore, detailed biophysical studies, including per-residue or segmental secondary structure formation properties and their role in peptide aggregation, are important for better understanding and, potentially, regulation of the amyloidogenic behavior of Aβ. On the other hand, identification of specific structural and aggregation propensities of defined stretches of Aβ and their effects on the behavior of the full-length Aβ_1–42_ may help develop peptide-based drugs to inhibit Aβ aggregation or immunotherapy approaches for Aβ clearance.

Such strategies of AD drug development have generated promising results. For example, DNA plasmid vaccine targeting Aβ N-terminus (Aβ_1–11_) elicited strong immune response and inhibited Aβ pathology in various animal models of AD^51–53^. The first potentially disease-modifying AD drug aducanumab was recently approved by the Food and Drug Administration based on its ability to clear Aβ from brain parenchyma. Short peptides, related or unrelated to Aβ sequence, have been employed to inhibit Aβ aggregation or disperse preformed oligomers and fibrils and impede Aβ-induced cytotoxicity^54–67^. The inhibitory effect of peptides on Aβ aggregation and toxicity stems from their ability to intercalate into Aβ assemblies and prevent toxic oligomer formation, which may or may not correlate with their own self-aggregation propensities. Thus, a mutated peptide, KLVFWAK, mimicking the Aβ sequence 16–22 (KLVFFAE), had low self-aggregation tendency yet preferentially interacted with oligomeric and fibrillar structures of Aβ^67^. The native sequence of Aβ_16–22_, on the other hand, has been identified as a fibrillogenic segment with high antiparallel β-sheet formation propensity^22,68,69^. Among several Aβ segments incorporated in a macrocyclic structure, the 16–22 segment inhibited Aβ_1–40_ aggregation and cytotoxicity^60^. These effects were interpreted in terms of binding of the fragment to Aβ oligomers and inhibition of fibril nucleation. Interestingly, the same 16–22 sequence, as well as the hydrophobic C-terminal stretch from Lys_28_ onward, were found to be the most fibrillogenic regions in the context of several Aβ species containing 38 to 43 amino acids^21^. Consistent with these findings, Aβ_28–42_ and Aβ_29–42_ displayed fast aggregation and β-sheet fibril formation propensities unlike some other C-terminal Aβ fragments^36^. Moreover, among twelve C-terminal Aβ_1–42_ fragments, Aβ_28–42_ exerted maximum toxic effect on cultured PC-12 cells^36^ and suggested correlation between amyloidogenicity and toxic effects of the peptides. Furthermore, two C-terminal fragments, Aβ_31–42_ and Aβ_39–42_, were able to rescue neuronal cells from Aβ_1–42_ cytotoxicity by interfering with formation of toxic oligomers^70^.

Thus, fragments of Aβ appear to affect the aggregation and toxicity of full-length Aβ by direct interaction with the latter. Studies on the details of binding of fragments to the full-length peptide revealed diverse modes of interaction. A C-terminal Aβ fragment Aβ_33–37_ (GLMVG) was shown to bind to the C-terminus of Aβ_1–42_, possibly the homologous sequence, and suppress its aggregation, neuronal membrane damage and synaptotoxicity^61^. Puzzlingly, another short C-terminal fragment, Aβ_39–42_, preferentially interacted with the N-terminus of Aβ_1–42_ in oligomeric form^71–73^. The aforementioned mutated peptide, KLVFWAK, also interacted in a non-homologous manner with the C-terminus of Aβ aggregates^67^.

These findings indicate that Aβ aggregation can be effectively modulated by Aβ-derived peptides. However, a complete understanding of the differential effects of Aβ segments along the entire Aβ_1–42_ sequence has not been achieved. This work aimed at systematic studies on 10 to 12 amino acid residue long overlapping fragments of Aβ_1–42_ (Fig. 1) to understand their intrinsic β-sheet formation and fibrillogenesis properties and their effects on same properties of the parent peptide. Out of seven peptide fragments, three stretches, corresponding to sequence 11–20, 26–36, and 31–42, have been identified that demonstrated strong β-sheet and aggregation propensity. The former two sequences were able to inhibit Aβ_1–42_ fibrillogenesis by up to 80%, whereas the C-terminal dodecapeptide, as well as the fragment 6–15, displayed moderate (20–30%) inhibitory potency. Further spectroscopic studies shed light on the mode of peptide-peptide interactions. Our data thus provide significant new information on intrinsic biophysical properties of Aβ_1–42_ segments and their effects on the behavior of the full-length peptide, which may be useful in designing peptide-based AD therapies.

## Results

### Choice of peptide fragments

Seven overlapping fragments of Aβ_1–42_ have been studied, i.e. sequences 1–10, 6–15, 11–20, 16–25, 21–30, 26–36, and 31–42, named P1, P2, P3, P4, P5, P6, and P7, respectively. All peptides have been N-terminally acetylated and C-terminally amidated to eliminate the charge effects of terminal amine and carboxyl groups. We chose to study overlapping peptides to avoid missing the effects of sequences at boundaries of consecutive segments. The locations of these seven fragments along the Aβ_1–42_ sequence and their respective theoretical isoelectric point (pI) values are presented in Fig. 1.

### Secondary structure from FTIR spectroscopy

The lyophilized peptides were dissolved in hexafluoroisopropanol (HFIP) to disperse possible pre-existing aggregates^74^. HFIP solution of the Aβ_1–42_ peptide was spread over a CaF_2_ disk, air-dried and desiccated for 15 min, followed by measurements of Fourier transform infrared (FTIR) spectra. The dry peptide acquired α-helical structure manifested by a sharp amide I peak at 1658 cm^−1^ (Figure S1 of Supplementary Information). Before cleavage from APP, most of the Aβ sequence corresponds to the transmembrane α-helix embedded in neuronal membranes^75^. Hence, this result is consistent with the intrinsic α-helical propensity of the Aβ_1–42_ sequence in dehydrated state. On the other hand, determining the structure of the dehydrated peptide is important for analysis of structural transitions once the peptide is exposed to an aqueous medium.

**Figure 2:**
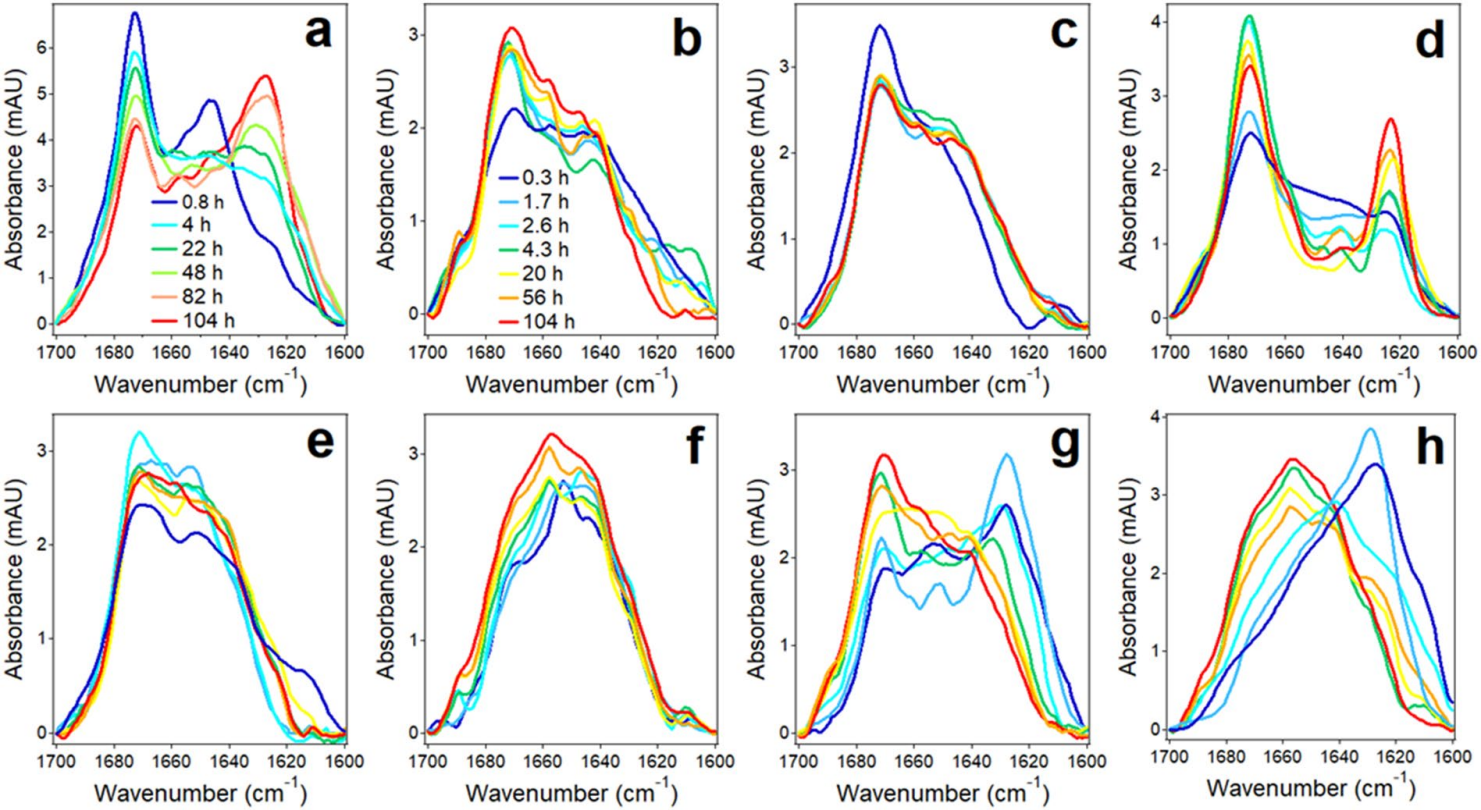
FTIR spectra at indicated times of incubation in D_2_O-based buffer (25 mM NaCl, 25 mM Na,K-phosphate, pD 7.2) for Aβ_1–42_ (**a**) and peptides P1 (**b**), P2 (**c**), P3 (**d**), P4 (**e**), P5 (**f**), P6 (**g**), and P7 (**h**) at 25 °C. The concentrations of the peptides were 50 μM for Aβ_1–42_ and 100 μM for the fragments. Times of incubation in buffer of all fragments are indicated in panel (**b**).

Next, HFIP solutions of Aβ_1–42_ and the seven fragments were separately dried in glass vials and aqueous (D_2_O) buffer was added to reach concentrations of 50 μM for Aβ_1–42_ and 100 μM for the fragments. The samples were constantly stirred with a small magnetic stir bar, and FTIR spectra of 70 μL aliquots were measured periodically over 100 h to examine structural changes of the peptides. The amide I spectrum of Aβ_1–42_ initially showed two prominent components at 1672 and 1647 cm^−1^, suggesting mostly β-turn and unordered or loop conformations^76–78^ (Fig. 2a). Upon incubation in buffer, the 1647 cm^−1^ peak was gradually replaced by a component at 1627 cm^−1^, indication formation of intermolecular β-sheet structure^76,78^. This is consistent with the fibrillar structure of Aβ_1–42_ comprising β-sheet and β-turn/loop conformations^32–34^ (Fig. 1) and indicates that formation of turn/loop structures precedes aggregation and fibrillogenesis through intermolecular H-bonding.

The fragments exhibited distinct structural transitions in aqueous buffer. All fragments except for P7 initially displayed significant fraction of β-turn structure in addition to irregular structure, indicated by amide I components in 1672–1670 cm^−1^ and 1640 cm^−1^ regions, respectively (Fig. 2). Among these 6 peptides, only P3 and P6 showed β-sheet component at 1628–1623 cm^−1^ (Fig. 2d, g). In case of P3, both β-turn and β-sheet structures grew at the expense of irregular structure (Fig. 2d), whereas in P6 the β-turn became the dominant structure upon prolonged incubation in buffer (Fig. 2g). P7 exhibited a unique behavior; it rapidly formed β-sheet structure with a distinctive peak around 1626 cm^−1^, which gradually shifted to higher wavenumbers possibly reflecting transition to turn and/or α-helix structures. Thus, peptides P3, P6, and P7 display β-sheet formation propensity with distinct dynamics of structural transitions whereas the other four peptides tend to adopt turn/loop conformations.

### Secondary structure from CD spectroscopy

The structural transitions of the peptides in aqueous medium were further analyzed by a complementary method, circular dichroism (CD). In the same time, the question was addressed as to how the structural transitions of the peptides are related to fibrillogenesis. This was accomplished by near-simultaneous measurements of CD spectra, light scattering, and thioflavin-T (ThT) fluorescence of same peptide samples in aqueous buffer for over 100 h. CD spectra were measured for peptides dissolved in HFIP, in dry state, and in aqueous buffer. The spectrum of the full-length Aβ_1–42_ peptide in HFIP displayed a deep minimum at 204 nm and a shoulder around 220 nm (Figure S2a of Supplementary Information), indicating the presence of type I and II β-turns, β-strand, and possibly α-helix conformations^79,80^. CD spectra of seven peptide fragments exhibited two negative ellipticities as well. The stronger component occurred in the 205–208 nm region (P1 and P2) or 195–200 nm region (P3 through P7) (Figure S2b–h of Supplementary Information), assigned to β-turn and unordered structures, respectively^81–83^. The weaker component was located between 220 and 230 nm, which can be assigned to α-helix, β-turn and β-strand conformations.

The solvent was removed by desiccation and CD spectra of dry peptides were measured. These spectra showed significant structural changes upon drying. Aβ_1–42_ adopted mostly α-helical structure, evidenced by a negative component at 223 nm and weaker one at 208 nm (Figure S2a of Supplementary Information), which are generated by *n*–π* and π–π* electronic transitions^80,83^. Similar spectral features were displayed by P1, P3, and P6. Weaker intensity of the π–π* transition can be attributed to a more flexible or distorted α-helix^80^. In spectra of peptides P4 and P5, the π–π* transition was stronger, suggesting formation of more stable α-helix. Dry peptides P2 and P7 showed just one component around 220–223 nm, which is most likely to represent type-I β-turn and β-strand structures^79,81^.

**Figure 3:**
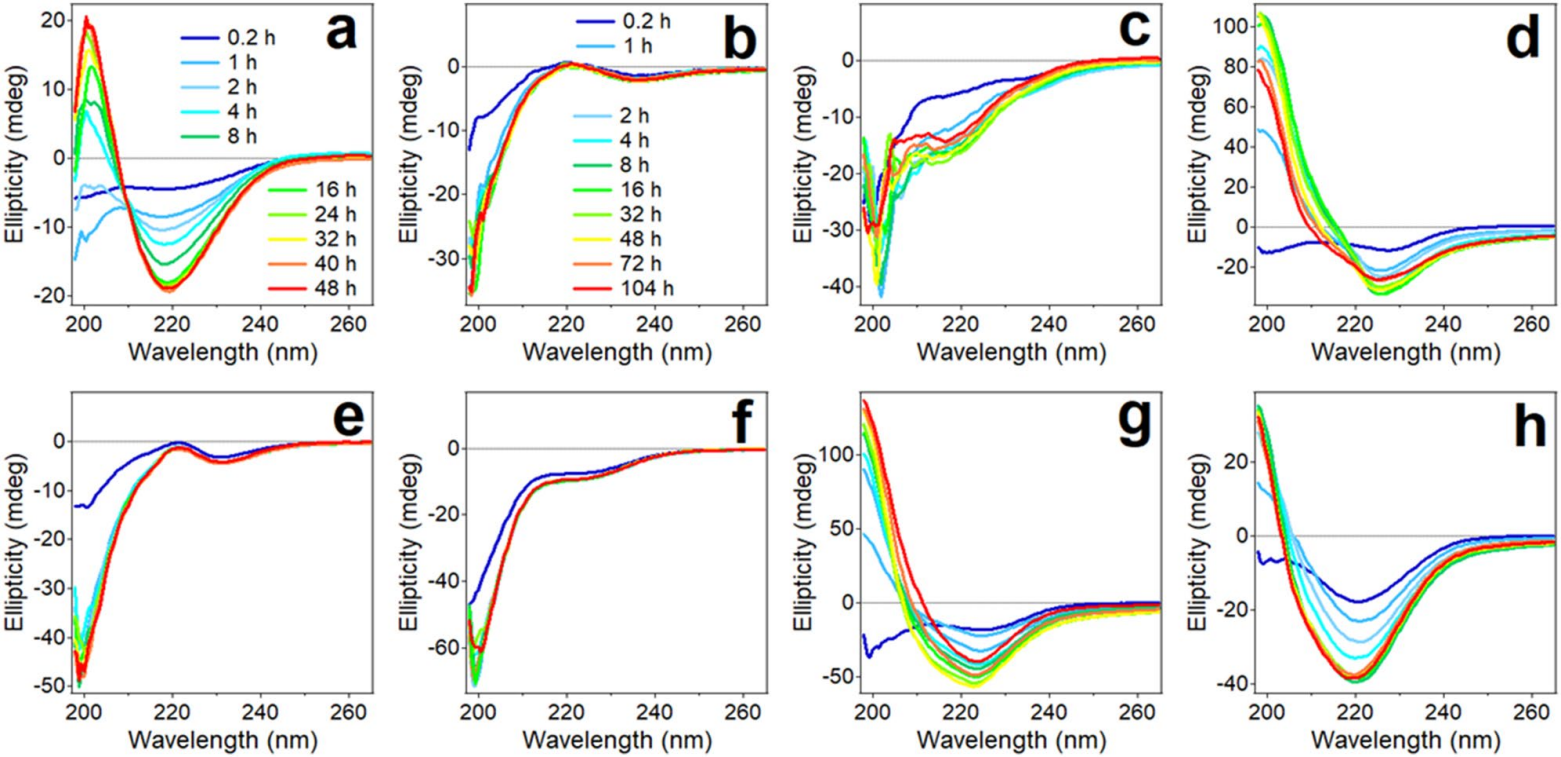
CD spectra of 50 μM Aβ_1–42_ (**a**) and 100 μM of peptides P1 (**b**), P2 (**c**), P3 (**d**), P4 (**e**), P5 (**f**), P6 (**g**), and P7 (**h**) constantly stirred by a tube rotator in buffer (25 mM NaCl, 25 mM phosphate, pH 7.2) for time periods indicated in panel a for Aβ_1–42_ and in panel (**b**) for all fragments. All measurement were conducted at 25 °C.

Following structural characterization of the peptides dissolved in HFIP and in dry state, aqueous buffer was added and structural transitions were followed by recording consecutive CD spectra. Spectra of Aβ_1–42_ initially showed a shallow negative band around 217 nm, which upon incubation in buffer for up to 48 h became a well-defined band accompanied with a positive component of nearly equal intensity around 200 nm, clearly indicating formation of β-sheet structure (Fig. 3a). Structural transitions in peptide fragments occurred at slower rate, therefore they were examined for longer time. Peptides P1, P2, P4, and P5 retained strong negative ellipticity around 200 nm and weaker components between 225 and 235 nm (217 nm for P2), implying these peptides assume mostly unordered conformation with fractions of β-turn/β-strand structures (Fig. 3b, c, e, f). In contrast, peptides P3, P6, and P7 readily (in less than 1 h) adopted defined secondary structures (Fig. 3d, g, h). Spectra of these three peptides display a negative *n*-π* band around 220–225 nm and strong positive ellipticity in the 200 nm region. These spectral features can be assigned to twisted β-sheet structure, as the intense π–π* transition is diagnostic for twisted β-strands, and the n–π* band can be significantly red-shifted depending on the degree of strand twist^80–82,84,85^. Note that CD spectra become excessively noisy below 200 nm due to the relatively long optical path-length (4 mm) of the cuvette, which was used for simultaneous light scattering and fluorescence measurements. However, this does not invalidate above interpretations of CD spectra in terms of peptides’ structural features.

Some differences have been observed between peptide structures deduced from FTIR and CD measurements, such as different relative contents of β-turn and irregular structures. These differences can be attributed to non-identical sample preparation and handling protocols, as Aβ structure is exquisitely sensitive to sample preparation conditions, including the method of sample stirring^24,32,86–88^. In addition, both N-terminal acetylation and C-terminal amidation introduce additional amide bonds that generate FTIR signal in the unordered region, i.e. between 1650 and 1638 cm^−1^. Nonetheless, both FTIR and CD data unveil important common structural trends of these peptides, i.e., P3, P6, and P7 stand out by exhibiting strong β-sheet propensity, like the parent peptide, while peptides P1, P2, P4, and P5 tend to stay in β-turn or unordered conformations.

### Aggregation and fibrillogenesis

**Figure 4:**
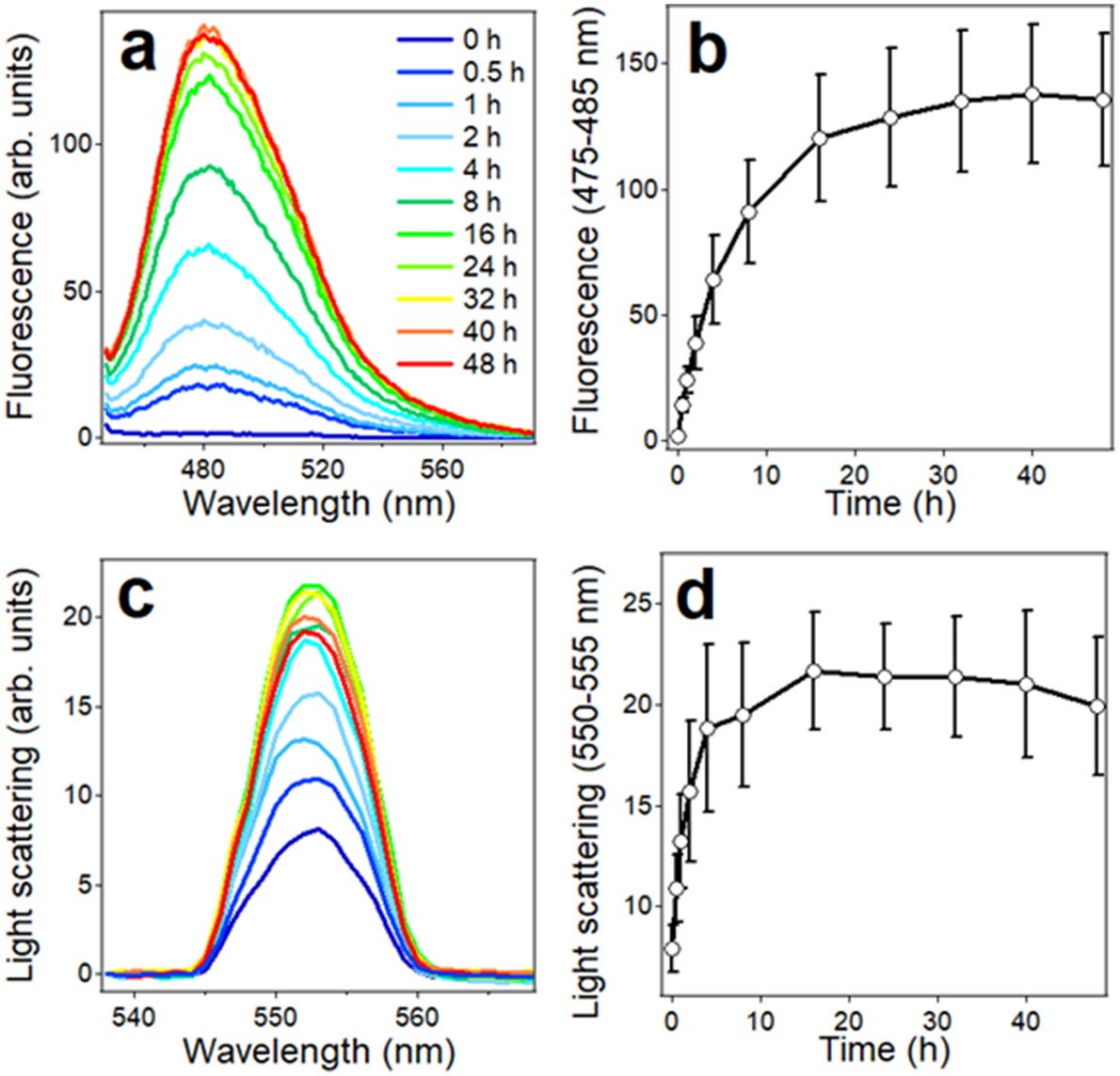
ThT fluorescence with excitation at 435 nm (**a**) and light scattering with incident light at 550 nm (**c**) of 35 μM Aβ_1–42_ in buffer (25 mM NaCl, 25 mM Na,K-phosphate, pH 7.2) stirred by a tube rotator for time periods indicated in panel a. Time dependence of ThT fluorescence averaged between 475 and 485 nm and of light scattering averaged between 550 and 555 nm are presented in panels (**b**) and (**d**), respectively, which represent average data and standard deviations from three independent experiments.

ThT fluorescence and static light scattering at 90 degrees were used to examine the kinetics of aggregation and fibrillogenesis of the peptides in aqueous buffer. Of note, these measurements were conducted in parallel with CD on same samples, which allowed analysis of structural changes, peptide aggregation and fibril formation in real time, excluding sample-to-sample errors. Increase in ThT fluorescence around 480 nm when excited at 435 nm was interpreted as fibril formation with an understanding that non-fibrillar structures could also bind ThT and produce fluorescence in that region. Aβ_1–42_ underwent fibrillogenesis without delay, as indicated by increase in ThT fluorescence around 480 nm (Fig. 4a, b). Saturation of ThT fluorescence was reached by around 1 day of incubation in buffer with constant stirring on a rotary mixer. Light scattering at 550 nm increased with steeper initial rate and approached saturation by 10–20 h of incubation (Fig. 4c, d). The difference in the initial kinetics of fluorescence and light scattering may reflect rapid formation of non-fibrillar aggregates that do not bind ThT.

**Figure 5:**
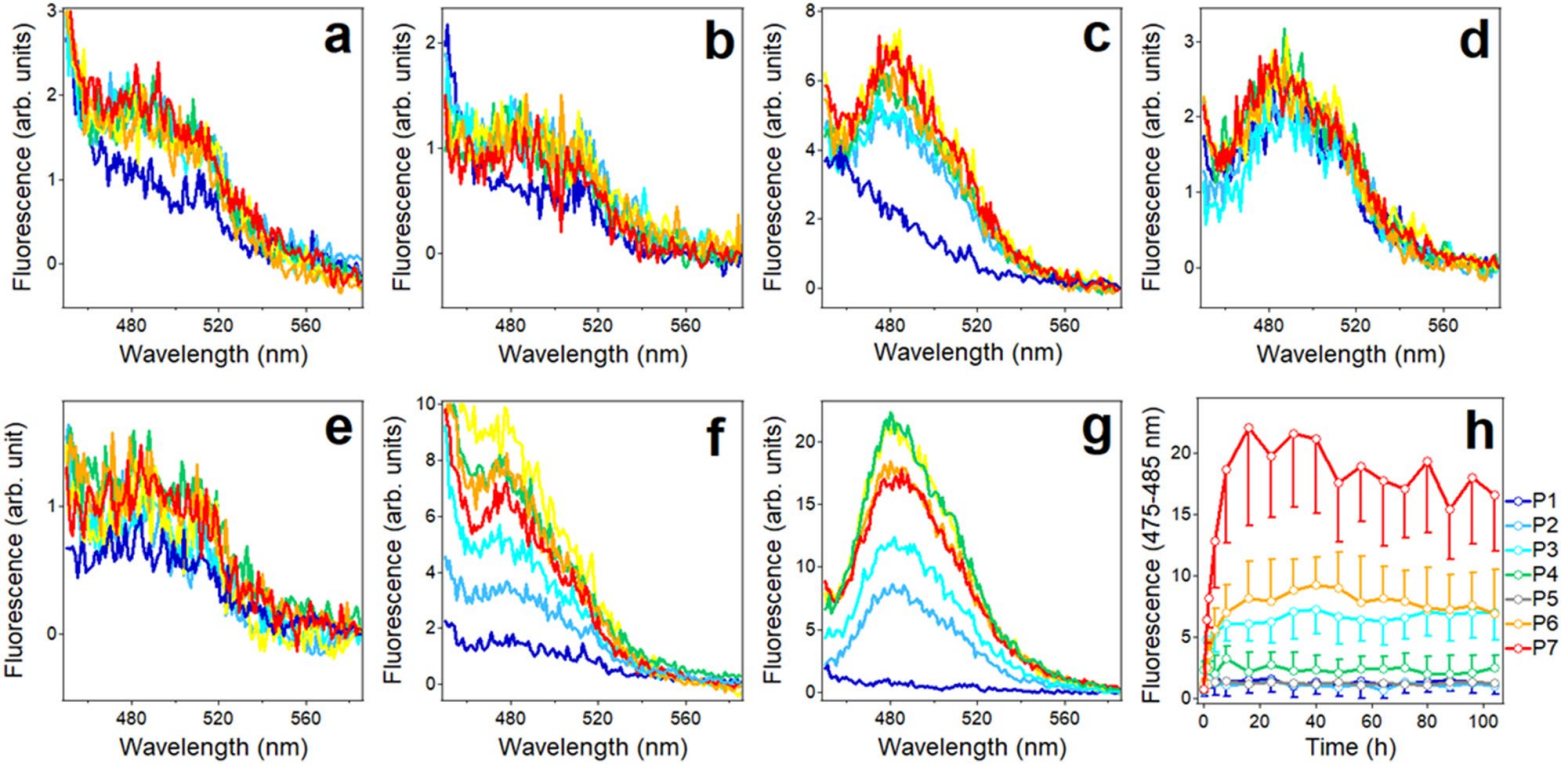
ThT fluorescence spectra of P1, P2, P3, P4, P5, P6, and P7 (panels a through g, respectively) with excitation at 435 nm, at 25 °C. Time of incubation of peptide samples in buffer (25 mM NaCl, 25 mM Na,K-phosphate, pH 7.2) at 70 μM concentration with constant stirring by a tube rotator is as follows: 0.1 h (dark blue), 2.0 h (light blue), 4.0 h (turquoise), 16.0 h (green), 40.0 h (yellow), 64.0 h (orange), 104.0 h (red). Panel h shows the time dependence of ThT fluorescence averaged between 475 and 485 nm for all seven peptides. Average data from three independent experiments. Error bars are shown only for P1, P3, P4, P6, P7 to maintain clarity. Error bars for P2 and P5 are of similar magnitude as for those of P1 and P4.

In samples with peptide fragments P1, P2, P4, and P5, very weak ThT fluorescence was detected (Fig. 5a, b, d, e). Peptides P3 and P6 generated higher level of ThT fluorescence, and peptide 7 produced maximum ThT fluorescence (Fig. 5c, f, g), unraveling a trend of fibrillogenesis propensity P7 > P6 ≈ P3 >> P4 ≈ P5 ≈ P2 ≈ P1 ≈ 0 (Fig. 5h). Statistical *t*-test analysis of all data of Fig. 5 between 24 and 104 h of incubation in buffer produced nearly 98% credibility for significant difference between fibrillogenesis propensities of P7 and P3 (*p* = 0.0214 ± 0.0084) and more than 96% credibility between P7 and P6 (*p* = 0.0385 ± 0.0101). P3 and P6 were not significantly different (*p* = 0.593 ± 0.241), and the difference between P3 and P1, P2, P4, P5 was 96.9–98.7% significant.

Concurrent light scattering measurements indicated relatively weak signal for peptides P1, P2, P4, and P5 and about threefold higher signal for peptides P3, P6, and P7 (Figure S3 of Supplementary Information). For the first group of peptides, *t*-test analysis produced *p* values between 0.172 ± 0.085 (P2/P5) and 0.747 ± 0.232 (P1/P4), and for the second group of peptides, *p* values varied between 0.366 ± 0.270 (P3/P7) and 0.685 ± 0.159 (P3/P6), indicating little statistical difference within each group. The difference between these two groups was significant (> 96%), as indicated by *p* values for P2/P3 of 0.0367 ± 0.0122. Negligible ThT fluorescence but appreciable light scattering of peptides P1, P2, P4, and P5 apparently reflects formation of non-fibrillar aggregates by these fragments. Higher level of ThT fluorescence by P7 compared to P3 and P6 but similar light scattering (cf. Fig. 5h and S3h) suggests a higher ThT binding capacity of P7, plausibly due to its exceptional hydrophobicity and/or a larger number of amino acid residues. Overall, these data provide evidence that peptides P3, P6, and P7 possess significant aggregation/fibril formation capability as compared to the other four Aβ fragments. In conjunction with structural data, it appears that the aggregation/fibrillogenesis property of these peptides correlates with their β-sheet formation ability.

### Effects of Aβ fragments on Aβ fibrillogenesis

**Figure 6:**
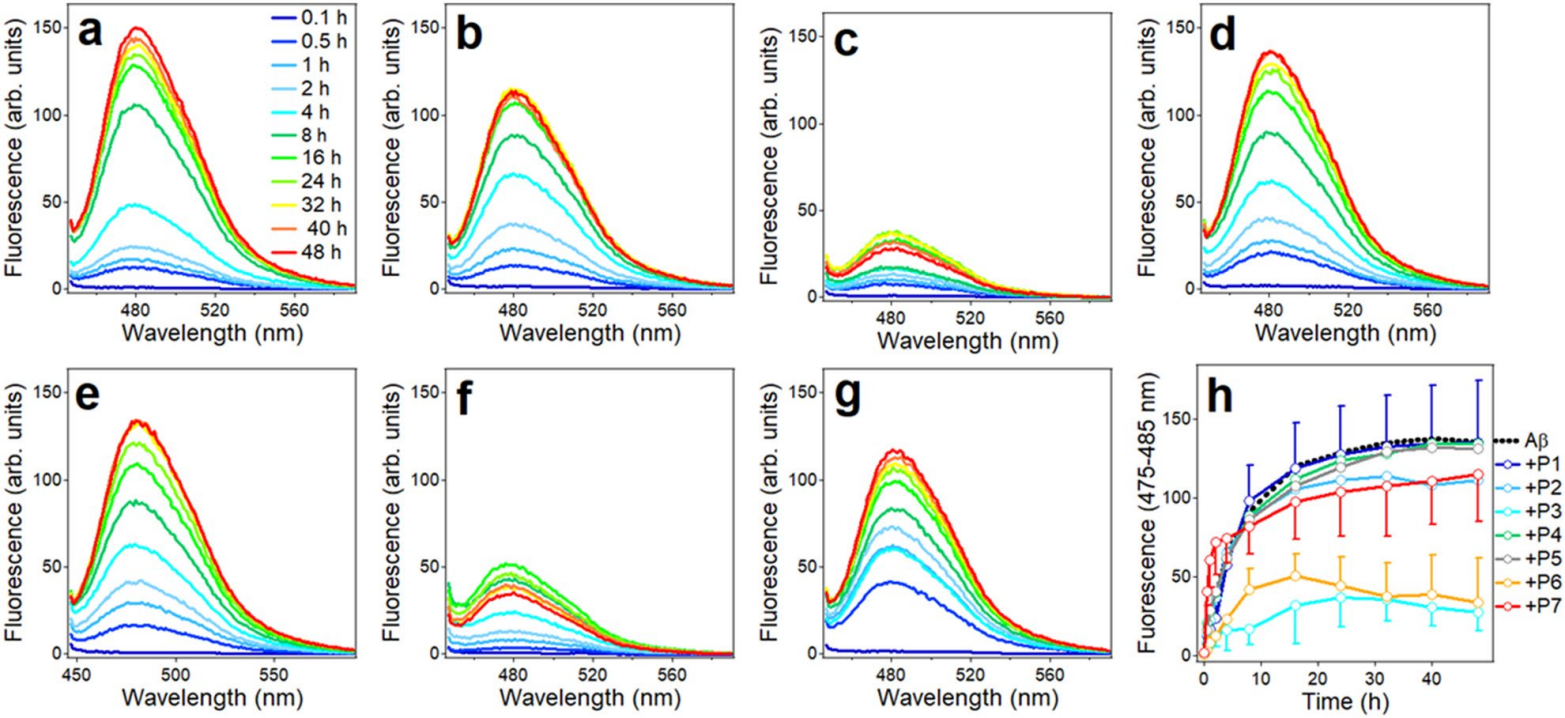
ThT fluorescence spectra of 35 μM Aβ_1–42_ plus 70 μM P1 (**a**), P2 (**b**), P3 (**c**) P4 (**d**), P5 (**e**), P6 (**f**), and P7 (**g**) with excitation at 435 nm, at 25 °C. Time of incubation in buffer (25 mM NaCl, 25 mM Na,K-phosphate, pH 7.2) with constant stirring by a tube rotator is indicated in panel (**a**). Vertical axes are standardized for better comparison of relative fluorescence intensities. Panel (**h**) shows the time dependence of ThT fluorescence averaged between 475 and 485 nm. Average data from three independent experiments. Error bars are shown only for P1, P3, P6, P7 to maintain clarity. Error bars for P2, P4, and P5 are of similar magnitude as for those of P1 and P7.

**Figure 7:**
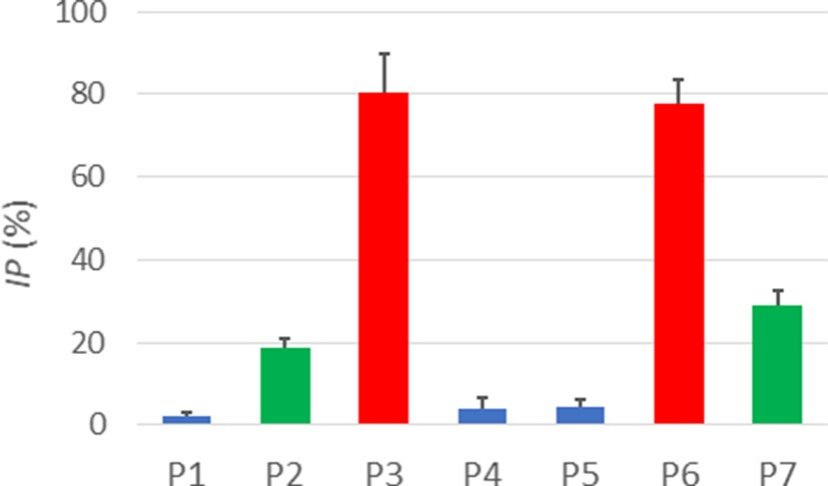
Inhibition percentages for P1 through P7 calculated based on ThT fluorescence data (Figs. 4, 5, 6) averaged between 32 and 48 h of incubation of Aβ_1–42_ alone and with each peptide fragment at twofold molar excess in buffer (25 mM NaCl, 25 mM Na,K-phosphate, pH 7.2). Statistical difference between bars of different colors is significant (*p* < 0.01). Difference between bars of same color is less significant or insignificant: for blue bars, *p* values vary between 0.113 and 0.851, for green bars *p* = 0.0163, and for red bars *p* = 0.673.

If some of peptide fragments demonstrate strong fibrillogenesis propensity, they might be able to intercalate into the assemblies of the parent peptide by binding to respective stretches and thereby interfere with its aggregation. To test this hypothesis, fibrillogenesis of Aβ_1–42_ was measured in the absence and presence of all seven peptide fragments at two-fold molar excess. In the presence of peptides P1, P2, P4, P5, and P7, ThT fluorescence of Aβ_1–42_ samples gradually increased and levelled off by 48 h of incubation with little effect of these fragments (Fig. 6a, b, d, e, g, h). In contrast, P3 and P6 strongly reduced the level of ThT fluorescence (Fig. 6c, f, h). Quantitative evaluation of inhibition of Aβ_1–42_ fibril formation by peptide fragments was performed by calculating the inhibition percentages (*IP*) for each fragment, as described in “Materials and methods”. P3 and P6 strongly inhibited Aβ_1–42_ fibrillization by 80.5 ± 9.0% and 77.7 ± 5.8%, respectively, with insignificant statistical difference between them (*p* = 0.673) (Fig. 7). Peptides P2 and P7 exhibited moderate inhibitory potencies (*IP* = 18.8 ± 2.1% and 28.7 ± 3.8%, respectively), with considerable statistical difference (*p* = 0.0163), and the other three peptides (P1, P4, and P5) were not potent inhibitors (*IP* < 5.0%; *p* values from 0.113 to 0.851). The difference between these three groups, presented by bars of distinct colors in Fig. 7, was statistically significant (*p* < 0.01).

### Mode of peptide interactions from Phe-to-Tyr energy transfer

The next question we explored was whether the intrinsic fluorescence of peptide fragments can be utilized to gain insight into the mode of their interactions. Aβ_1–42_ is devoid of tryptophan, but has one tyrosine (Tyr) at position 10 and three phenylalanines (Phe) at positions 4, 19, 20. Phe to Tyr resonance energy transfer (RET) has been described earlier for a tryptophan-less protein, with Förster distance of *R*_0_ = 13.5 Å^89,90^, which is much shorter than that of many other donor–acceptor pairs, typically in the 20 Å to 60 Å range^90^. This provides a unique opportunity to examine the interactions between peptides containing Phe and Tyr by RET.

As both Tyr and Phe can be excited in a wide wavelength range^90^, an optimal wavelength needs to be identified where Phe absorbs maximally and Tyr absorbs minimally. Our measurements showed that this wavelength range was around 220 nm. Figure S4 of Supplementary Information shows how RET works for the P3/P1 donor/acceptor pair. Upon excitation at 220 nm, Phe and Tyr of peptides P3 and P1 emit around 287 and 310 nm, respectively (Figure S4a,b). In equimolar mixture of these peptides at same total peptide concentration, the intensity of Phe emission gradually decreases, indicating energy transfer to Tyr. Negative change of Phe fluorescence (DF_Phe_ < 0) and positive change in Tyr fluorescence (DF_Tyr_ > 0) are clearly seen in difference spectra around 30 h of incubation of P1 and P3 (Figure S4c), indicating efficient RET.

**Figure 8:**
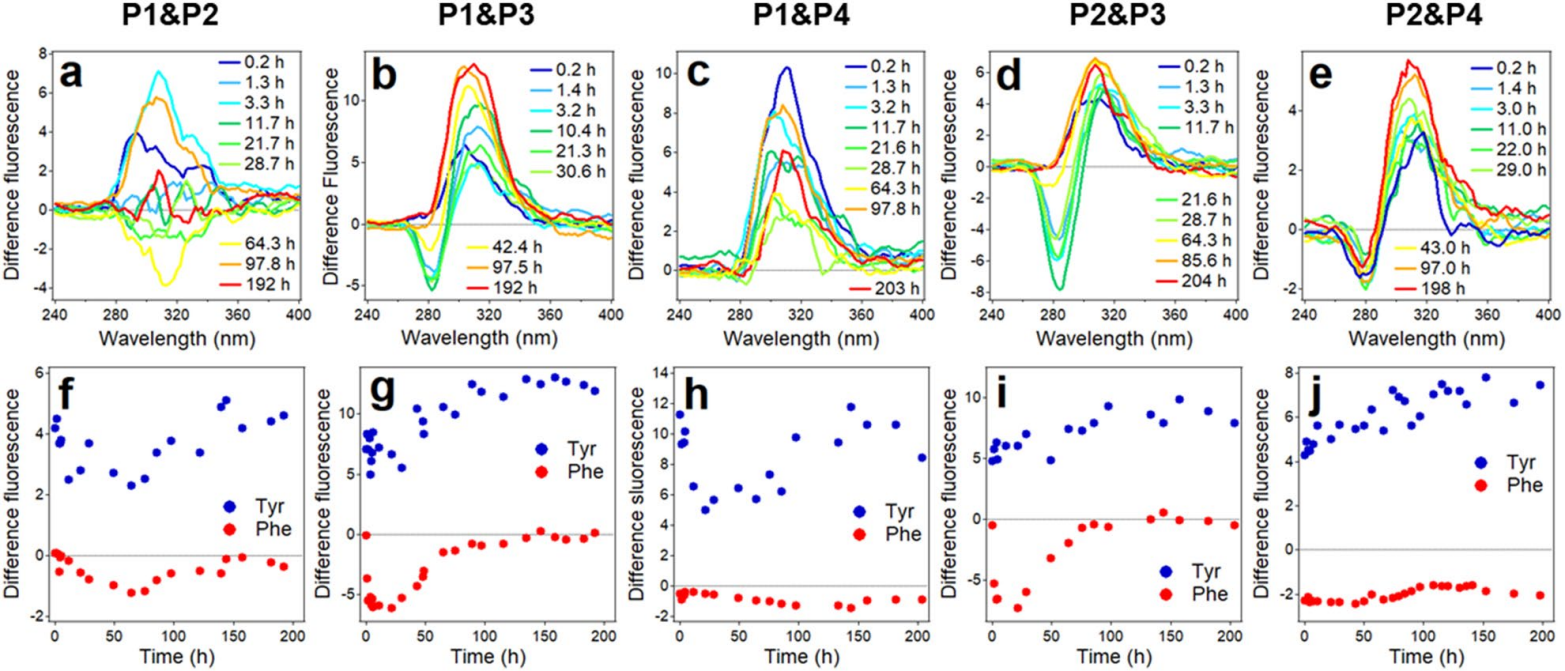
Fluorescence resonance energy transfer difference spectra (**a** through **e**) and values for tyrosine and phenylalanine, averaged between 300 and 320 nm for tyrosine and 270 and 290 nm for phenylalanine (**f** through **j**) for five peptide systems, as indicated at the top. Fluorescence spectra have been smoothed by 13-point Savitzky–Golay least-squares polynomial algorithm before subtraction. See details in “Materials and methods”.

As seen from Fig. 1, only peptide fragments P1, P2, P3, and P4 contain Phe and/or Tyr fluorophores, hence these four peptides have been analyzed in RET experiments. Figure 8 shows RET data for five peptide pairs that satisfy this criterion, i.e. possibility of Phe-to-Tyr RET. These data indicate slightly negative DF_Phe_ in P1/P2 and P1/P4 systems, implying little RET in these peptide samples (Fig. 8a, c, f, h). Significant RET is seen in combinations of P3 with P1 and P2; in both cases negative DF_Phe_ develops soon after mixing the peptides, reaches maximum values between 10 and 30 h, and returns to zero level between 50 and 100 h of incubation in buffer (Fig. 8b, d, g, i). In P2/P4 combination, there is relatively moderate RET (DF_Phe_ < 0) for the whole time period of the experiment, i.e. ~ 200 h (Fig. 8e, j), indicating stable hetero-aggregation between peptides P2 and P4. Values of DF_Tyr_ are always positive, consistent with RET; their variations can be attributed to quenching effects of water and/or carbonyl groups of peptides during the aggregation process, as described earlier^71^.

These data indicate that P3 not only readily self-aggregates, as seen from ThT fluorescence and light scattering data (Figs. 5h and S3h), but also effectively interacts with non-homologous stretches of Aβ, such as P1 and P2 segments, pointing to the “sticky” nature of P3. In the P1/P3 system, the peptides are likely to interact in parallel in-register manner, positioning Tyr_10_ against Phe_20_ (Figure S5a of the Supplementary Information). Strong RET between P2 and P3 suggests parallel out-of-register pairing, with Phe_19_ facing Tyr_10_ (Figure S5n of the Supplementary Information). These interactions apparently start falling apart at the end of first day of incubation (Fig. 8g, i), as P3 predominantly forms homologous aggregates, as described below. Data of Fig. 8e, j suggest that P2 and P4 form persistent aggregates, presumably of parallel out-of-register sense allowing juxtaposition of Phe_19_ and Tyr_10_ (Figure S5c of the Supplementary Information). It is interesting to note that in these proposed models of peptide interactions, ionic bonds between side chains of Asp_7_ and Lys_16_ play an important role by stabilizing hetero-aggregation in both P2/P3 and P2/P4 systems (Figure S5b,c of the Supplementary Information).

To gain insight into the mode of interaction of P3 with Aβ_1–42_, RET experiments have been conducted with this peptide system. Fluorescence spectra were measured for 35 μM Aβ_1–42_, 70 μM P3, and 35 μM Aβ_1–42_ + 70 μM P3 over a time period of 48 h, with excitation at 220 nm (Figure S6 of the Supplementary Information). First, we checked if RET effect was involved in self-aggregation of Aβ_1–42_ by subtracting the spectrum of Aβ_1–42_ measured at 1 h from spectra measured at 2 h and beyond. These difference spectra showed time-dependent changes in Tyr fluorescence, reflecting at least two effects, i.e., suspension of the dry peptide into the aqueous medium (decrease of fluorescence because of quenching by water) and sequestration from the aqueous medium through fibrillogenesis (increase of fluorescence), but a combination of negative ΔF_Phe_ and positive ΔF_Tyr_ has not been detected (Figure S6d of the Supplementary Information). This means absence of RET between Aβ_1–42_ molecules, consistent with formation of parallel, in-register β-sheet aggregates. A similar procedure for the Aβ_1–42_ + P3 system, as well as subtraction of spectra of both Aβ_1–42_ and P3 from those of the combined sample at respective times, yielded RET effect at 32–48 h of co-incubation, i.e. DF_Phe_ < 0 and DF_Tyr_ > 0 (Figure S6e,f of the Supplementary Information). This outcome implies that during interaction of P3 with Aβ_1–42_, Phe_19_ and Phe_20_ of at least a fraction of P3 come to close proximity to Tyr_10_ of Aβ_1–42_ at 32–48 h. However, the inhibitory effect of P3 on Aβ_1–42_ fibrillogenesis starts at earlier times (Fig. 6h), indicting that P3 interacts more effectively with other regions of Aβ_1–42_, which need to be identified in further studies.

## Discussion

This work reports an analysis of structural and aggregation propensities of Aβ_1–42_ fragments and their effects on Aβ_1–42_ fibrillogenesis. The results facilitate a better understanding of segment-specific properties of Aβ_1–42_ and identify peptide fragments capable of inhibiting its aggregation. We chose 10- to 12-residue overlapping fragments because fragments shorter than 10 residues might be unable to exhibit robust secondary structure formation and fibrillogenesis abilities because of insufficient length. Although short Aβ fragments containing 6–8 residues have been shown to adopt parallel or antiparallel β-sheets in crystal form^91,92^, the hydrophobic C-terminal sequences of Aβ_1–42_ containing eight amino acid residues, as well as an 11-residue stretch (Aβ_32–42_), did not aggregate into β-sheet fibrils in aqueous buffer even at concentrations exceeding 100 mM^36^. On the other hand, use of overlapping sequences was important for characterization of segments at boundaries of consecutive stretches; properties of the boundary between P(*i*) and P(*i* + *2*) were reflected in P(*i* + *1*), which covers the C- and N-termini of flanking sequences.

Peptides P1 and P2 represent the N-terminus of Aβ, which has been consistently found in unordered conformation in the context of Aβ_1–40_ or Aβ_1–42_^24,31,32,34,86,93^. The Aβ_1–8_ fragment was found in extended coil confirmation in complex with a monoclonal antibody^94^. Despite its proposed role in stabilizing Aβ fibrils^95^, above data and our findings indicate that the N-terminus of Aβ is intrinsically unordered and unable to undergo fibrillogenesis. Consistent with this, RET data indicate that P1 and P2 do not form hetero-aggregates as well (Fig. 8a, f). This feature is apparently related with the amino acid composition of these segments; the paucity of nonpolar side chains and the excess negative charge (pI 4.54 and 5.72 for P1 and P2, respectively) may prevent aggregation due to electrostatic repulsion and lack of hydrophobic interactions. P1 is also a poor inhibitor of Aβ_1–42 _aggregation, whereas P2 demonstrates moderate inhibitory activity (Fig. 7), which probably stems from its C-terminal part, as P3 is highly aggregation prone and a potent inhibitor of Aβ_1–42_ fibrillogenesis.

The P3 segment corresponds to one of the two major β-strands of Aβ_1–40_ fibrillar structure Aβ_1–42_^24,31,86^ (Fig. 1). The whole P3 sequence or at least its central part are in β-strand conformation in fibrils formed by of Aβ_1–42_ as well^32–34^ (Fig. 1). P3 has one glutamic acid (Glu) and one lysine (Lys) residues and nearly zero net charge (pI = 7.01). These features would allow the peptide to form β-strand-like non-H-bonded out of register aggregates in antiparallel sense with oppositely charged side chains facing each other, as shown in Figure S7 of the Supplementary Information. This arrangement will juxtapose one Glu-Lys pair and one histidine pair between strands so the structure will be stabilized by Glu-Lys salt bridges and π-π stacking interactions between histidines^96,97^. As a fraction of imidazole side chains will be protonated due to the close-to-neutral pK = 6.3 ± 0.1^98^, His-His attraction will be further strengthened by nucleophile–electrophile interactions. This structure may grow laterally beyond tetramer until decreasing internal energy is compensated by decreasing entropy at a minimum of free energy. The fibril will grow by means of parallel β-sheet formation stabilized by H-bonding perpendicular to the plane of side chains. The nonpolar overhangs may form turn or parallel β-sheet structure stabilized by hydrophobic interactions and π–π stacking interactions between C-terminal phenylalanines^96^. These conjectures are consistent with earlier observations of dimer formation by Aβ_10–23_ peptide that grow into β-sheet fibrillar structure^99^. FTIR data (Fig. 2d) show a low frequency (1623 cm^−1^) β-sheet component without a high-frequency counterpart diagnostic for antiparallel β-sheet, consistent with parallel β-sheet formation. Figures 5, 6, and 7 show that P3 forms fibrils and substantially blocks the fibrillogenesis of Aβ_1–42_. Inhibition of Aβ_1–42_ fibrillogenesis presumably occurs through intercalation of P3 aggregates between Aβ_1–42_ molecules and/or binding of P3 to the homologous or other segments of Aβ_1–42_. Heterologous binding is supported by the ability of P3 to effectively interact with P1 and P2 (Figures S5 and 8b, d, g, i). Consistent with our findings, it was reported earlier that Aβ segment 11–17 (EVHHQKL), i.e. 0.7 part of P3, but not other Aβ-derived short peptides, effectively blocked Aβ_1–40_-induced Ca^2+^ influx into cultured cells possibly by inhibiting membrane pore structure formation^58^.

Aβ region corresponding to P4 (Aβ_16–25_) contains the central hydrophobic core 17-LVFFA-21 that is deemed to be important for Aβ aggregation. Molecular modeling and experimental studies have shown that the Aβ_16–22_ fragment (KLVFFAE) forms antiparallel in-register β-sheet stabilized by nonpolar contacts between the central segment and ionic interactions between the side chains of N- and C-terminal Lys and Glu residues^22,68,69,85,100–102^. Monomeric Aβ_16–25_ was in coil or extended β-strand-like conformation and transitioned into antiparallel β-sheet through intermediate α-helical structure^103^. Our data show unordered plus β-strand conformation for P4 in HFIP in monomeric form (Figure S2e) but transition to β-sheet in aqueous buffer is not observed (Figs. 2e and 3e). This difference apparently results from the additional three residues in P4 compared to Aβ_16–22_. The presence of an additional anionic side chain of aspartic acid at position 23 results in excess negative charge and a low pI of 4.37 (Fig. 1). These considerations suggest that long-range electrostatic repulsion between short peptides may interfere with initial peptide aggregation and subsequent fibrillization. Furthermore, an analogue of Aβ_16–22_ with N-methyl amino acids in alternating positions was a potent inhibitor of Aβ_1–40_ fibrillogenesis unlike the unmodified peptide^55^, which is consistent with our data of negligible inhibitory effect of P4 on Aβ_1–42_ fibril formation (Figs. 6h and 7).

P5 encompasses the Aβ region that forms a loop between two major β-strands of Aβ_1–40_ fibrils characterized by two- or three-fold symmetry^24,31,86^ and has been viewed as a folding nucleus for Aβ^104,105^. This region is mostly in loop conformation in fibrils of Aβ_1–42_ as well^32–34^ (Fig. 1). In complex with an affibody protein, Aβ_1–40_ was found in a β-hairpin conformation with residues 24–29 forming a loop between two β-strands but without the Asp_23_-Lys_28_ salt bridge present in Aβ_1–40_ fibrils of two-fold symmetry^106^. The isolated P5 segment itself was mostly unordered^36^ or formed a loop with a central β-turn structure formed by residues 24 and 27 and stabilized by ionic interaction of Lys_28_ with Glu_22_/Asp_23_ and nonpolar contacts between Val_24_ and Lys_28_^104^. P5 did not undergo aggregation in aqueous medium^36,104^, in accord with sequence-based prediction of low aggregation propensity^21^. Transmission electron microscopic studies revealed relatively large (25–120 nm) globular particles formed by P5 with uncapped termini whereas the N-acetylated/C-amidated peptide formed smaller particles, implying low aggregation tendency^105^. Our FTIR data for P5 display a broad amide I band dominated by signal in the1670–1640 cm^−1^ range (Fig. 2f), revealing mostly unordered/turn structure of the peptide. This is confirmed by CD spectra with a deep minimum around 200 nm and a shallow negative band around 228 nm (Fig. 3f). Figure S3e,h and 5e, h demonstrate negligible light scattering and ThT fluorescence in P5 peptide samples, and Figs. 6h and 7 document the inability of P5 to affect Aβ_1–42_ fibrillogenesis, implying a poor potency of P5 towards homo- or hetero-aggregation.

P6 (Aβ_26–36_) resembles the Aβ_25–35_ peptide, which has been studied extensively due to its highly cytotoxic nature. In aqueous buffer at pH 4.0–5.5, the Aβ_25–35_ peptide underwent concentration-dependent unordered-to-β-sheet transition whereas its C-amidated counterpart was in random coil state^107^. At close to neutral pH, the N-acetylated/C-amidated peptide acquired β-sheet/β-turn structures^108^. In detergent micelles and in HFIP/water environment, Aβ_25–35_ was α-helical in the C-terminal region and less ordered or turn conformation in the N-terminus^109,110^. ThT and transmission electron microscopy assays revealed fibril formation by Aβ_25–35_^59,61^, and β-sheet breaking short peptides inhibited fibrillogenesis and cytotoxicity of the peptide^59^. Slight sequence differences in this region of Aβ resulted in significant differences in structural and aggregation properties. Biophysical and computational studies produced Aβ_26–36_ and Aβ_25–35_ structural models as β-hairpins containing two short β-strands separated by a turn^111^, in accord with our FTIR data (Fig. 2g). P6 formed β-sheet structure in aqueous buffer with red-shifted (~ 220 nm) n-π* CD band and exhibited higher fibril formation capability than similar undecapeptides Aβ_25–35_ and Aβ_24–34_^111^, consistent with our results (Figs. 3g, 5h).

P7 (Aβ_31–42_) represents the most hydrophobic C-terminal stretch of Aβ and plays an important role in its aggregation into β-sheet oligomers and fibrils^35,36,70^. Dynamic light scattering experiments identified two populations of Aβ_1–42_ aggregates with hydrodynamic radii (*R*_H_) 8–12 nm and 20–60 nm, which upon prolonged incubation in buffer exceeded 500 nm, while the P7 fragment formed large aggregates with *R*_H_ around 100 nm^36,70^. In combination, P7 affected the aggregation of Aβ_1–42_ in a nontrivial way; it increased the abundance of larger particles but reduced the rate of their formation. Molecular modeling simulations showed that P7 intercalates into Aβ_1–42_ aggregates and thereby inhibits formation of toxic assemblies of Aβ_1–42_^70^. CD experiments showed that structural transitions of C-terminal fragments of Aβ_1–42_ were concentration dependent; at 62 μM, P7 gradually transitioned from unordered state to β-sheet structure during 96 h of incubation in buffer and formed fibrils^36^, in good agreement with our data (Figs. 3h and 5g, h).

In conclusion, this work thoroughly characterizes the intrinsic aggregation and structural properties of overlapping peptide segments throughout the Aβ_1–42_ sequence and the effects of these fragments on Aβ_1–42_ fibrillogenesis. Although previous studies have utilized a similar approach and have focused on certain parts of Aβ_1–42_, such as the C-terminal hydrophobic stretch, systematic dissection and analysis of the whole Aβ_1–42_ sequence has not been reported. Or data identify Aβ fragments that are capable of self-aggregation and β-sheet structure formation and substantially inhibit fibrillogenesis of Aβ_1–42_, presumably by intervention into forming aggregates or prevention of efficient intermolecular contacts between Aβ_1–42_ molecules. However, there is no universal correlation between self-aggregation/β-sheet propensities of the fragments and suppression of Aβ_1–42_ fibril formation. For example, P7, the most hydrophobic segment that undergoes efficient fibrilization (Fig. 5h) and β-sheet formation (Fig. 2h and 3h) is a relatively week inhibitor of Aβ_1–42_ fibrillization of (Figs. 6h and 7). This leads to a conclusion that peptide fragments with high homo-aggregation propensity prefer to self-associate instead of forming hetero-aggregates with the full-length peptide. These findings, specifically the potent inhibitory effects of P3 and P6 peptides on Aβ_1–42_ aggregation, may help develop new DNA-, mRNA- or peptide-based anti-AD therapeutic strategies.

## Materials and methods

### Materials

The synthetic Aβ_1–42_ peptide was from Innovagen (Lund, Sweden) and was > 98% pure. Synthetic peptide fragments, acetylated at N-terminus and amidated at C-terminus, were purchased from Peptide 2.0 Inc. (Chantilly, VA, USA) and were 98–99% pure, as verified by high performance liquid chromatography and mass-spectrometry. HFIP, ThT, salts, buffers, and most of other reagents were purchased from Sigma-Aldrich (St. Louis, MO, USA) or Fisher Scientific (Hanover Park, IL, USA). Quartz cuvettes for CD measurements were from Starna Calls, Inc. (Atascadero, CA, USA). CaF_2_ windows for FTIR studies were from Buck Scientific (East Norwalk, CT, USA). D_2_O (99.8% pure) was from Cambridge Isotope Laboratories (Andover, MA, USA).

### Experimental procedures

#### Circular dichroism and fluorescence

Circular dichroism (CD) and fluorescence spectra were measured using a J-810 spectropolarimeter equipped with a fluorescence attachment and a temperature controller (Jasco, Tokyo, Japan) at 25 °C. Lyophilized peptides were dissolved in HFIP at a desired concentration and CD spectra were measured using a 4 mm × 4 mm rectangular quartz cuvette. Then, the solvent was removed by desiccation and CD spectra of the dry peptide stuck on the walls of the cuvette were measured. This was followed by addition of aqueous buffer (25 mM NaCl, 25 mM Na,K-phosphate, 20 μM ThT, pH 7.2) to achieve a target peptide concentration. The cuvette was capped with a Teflon stopper, sealed with parafilm, and rotated on a Fisherbrand™ Multi-Purpose Tube Rotator for several days. CD, ThT or intrinsic peptide fluorescence, and light scattering at 90 degrees were measured periodically on same sample, at pre-determined time pints, to follow the time dependence of structural changes and aggregation/fibrillogenesis of the peptides, using the multifunctional J-810 instrument. Measurements of these parameters on same samples in real time excluded sample to sample errors. For each peptide system, time-dependent spectroscopic measurements were repeated in three or four independent experiments.

ThT fluorescence was averaged between 475 and 485 nm, when excited at 435 nm, and plotted against time of incubation in buffer. For fluorescence RET experiments, incident light at 220 nm was used for predominant excitation of phenylalanine as compared to tyrosine, and spectra were measured between 200 and 400 nm. For each donor/acceptor peptide pair, the fluorescence of individual peptides was measured at 100 μM concentration, then spectra were recorded for the two peptides mixed at 50 μM each to maintain the same total peptide concentration. To determine the presence of RET, spectra of each peptide were subtracted from the spectrum of the combination multiplied a factor of 2. Reduction of the donor (Phe) but not acceptor (Tyr) fluorescence in the combination indicated RET effect. For light scattering, incident light at 550 nm was used to avoid generation of fluorescence signal and scattered light intensity at 90 degrees was measured between 530 and 570 nm. The signal intensity was averaged between 550 and 555 nm and plotted as a function of time.

The degree of inhibition of fibrillization of Aβ_1–42_ by peptide fragments was evaluated based on ThT fluorescence data. Total saturating ThT fluorescence between 32 and 48 h in the presence of both Aβ_1–42_ and a given fragment was considered as a sum of fluorescence due to each component. Then, fluorescence owing to Aβ_1–42_ fibrils in the presence of a fragment, F*, was determined by subtracting the signal measured for the fragment alone from total fluorescence. Fluorescence signal measured for pure fragments was corrected for concentration before subtraction, i.e. multiplied by 0.7, assuming a linear relationship between fluorescence and peptide concentration. Inhibition percentage of Aβ_1–42_ fibrillogenesis was calculated as *IP* = (1 − F*/*F*_0_) × 100%, where *F*_0_ is ThT fluorescence of Aβ_1–42_ in the absence of fragments.

#### FTIR spectroscopy

HFIP solution of the peptide was placed in small glass vials and the solvent was removed by desiccation. D_2_O-baseb buffer containing 25 mM NaCl and 25 mM Na,K-phosphate, adjusted to pD 7.2, was added to the dry peptide to achieve 50 μM concentration for the full-length peptide Aβ_1–42_ and 100 μM for the fragments. Peptide samples were continuously stirred by a small (3 mm in diameter) magnetic stir bar for > 100 h. At defined time points, a 70-μL peptide sample was placed between two CaF_2_ disks (32 mm in diameter, 3 mm thick), separated by a 50 μm thick Teflon spacer, and FTIR spectra were measured at 2 cm^−1^ nominal resolution, using a Vector-22 FTIR spectrometer (Bruker Optics, Billerica, MA, USA) equipped with a liquid nitrogen-cooled Hg-Cd-Te detector. Transmittance spectra of the blank buffer were measured and used as reference to calculate the absorbance spectra. For measurements of FTIR spectra of dry peptide sample, HFIP solution of the peptide was spread on a CaF_2_ disk, dried for 15 min in a desiccator, followed by measurement of the transmittance spectrum. A transmittance spectrum of the clean CaF_2_ disk was used as reference to calculate the absorbance spectrum. The instrument was constantly purged with dry air to avoid contribution of H_2_O vibrational/rotational features in the amide I region. Absorbance spectra of atmospheric H_2_O were measured separately and subtracted from spectra of peptides when such contribution was detected.

## Supplementary Information

Supplementary Information.
