# Supplementary material for: Effects of Aβ-derived peptide fragments on fibrillogenesis of Aβ
Source: Sci Rep. 2021 Sep 28;11:19262. doi: 10.1038/s41598-021-98644-y (PMC8479085; doi:10.1038/s41598-021-98644-y)
Supplement: Supplementary file 1 — Supplementary Information. [file 41598_2021_98644_MOESM1_ESM.docx]

**Supplementary Information**

**Effects of Aβ-derived peptide fragments on fibrillogenesis of Aβ**

Faisal Abedin^1^, Nabin Kandel^1,†^, and Suren A. Tatulian^2^*

1 Physics Graduate Program, University of Central Florida, Orlando, FL, USA

2 Department of Physics, College of Sciences, and Burnett School of Biomedical Sciences, College of Medicine, University of Central Florida, Orlando, FL, USA

† Current address: Center for Biotechnology and Interdisciplinary Studies, Rensselaer Polytechnic Institute, Troy, NY, USA

* Correspondence should be addressed to S.A.T. (Email: statulia@ucf.edu; Fax: 407-823-5112; Tel: 407-823-1543)


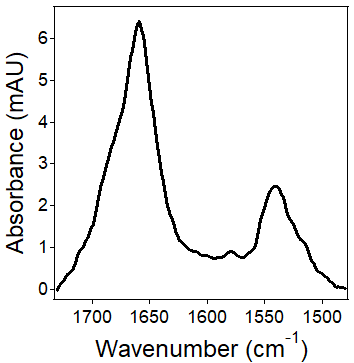


**Figure S1.** FTIR spectrum of Aβ_1-42_ dried on a CaF_2_ disk from 50 μM HFIP solution. Amide I and amide II peaks are located around 1657 cm^-1^ and 1537 cm^-1^, respectively.


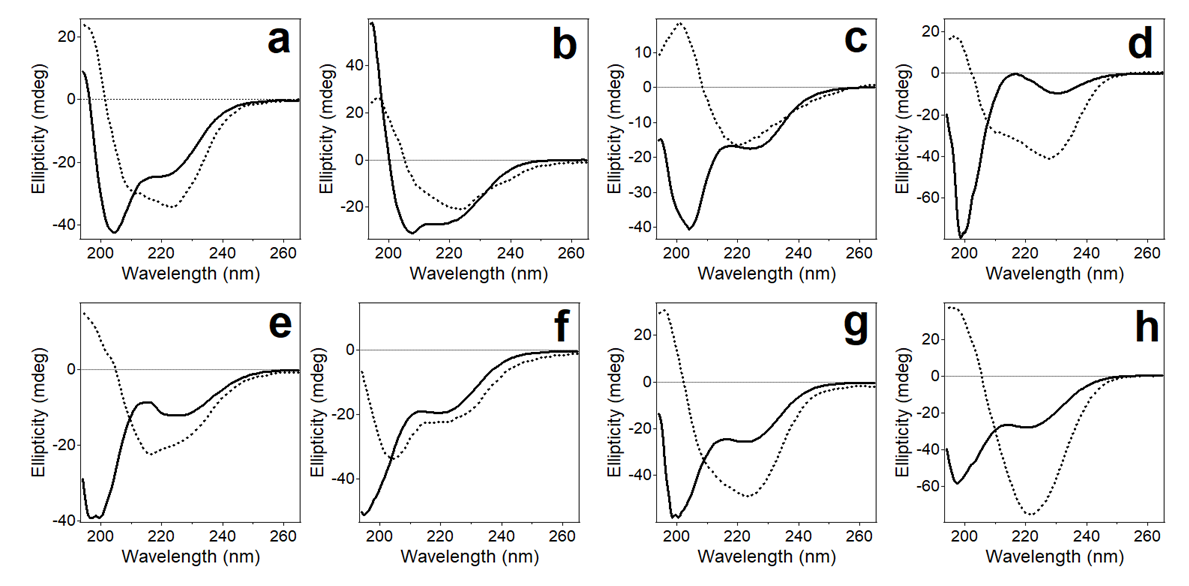


**Figure S2.** CD spectra of Aβ_1-42_ (a), P1 (b), P2 (c), P3 (d), P4 (e), P5 (f), P6 (g), and P7 (h) dissolved in HFIP (solid) and in dry state (dotted). The concentration of Aβ_1-42_ in HFIP is 35 μM and that of the fragments is 100 μM. The spectra of dry peptides are scaled up by a factor of 5 for better comparison.


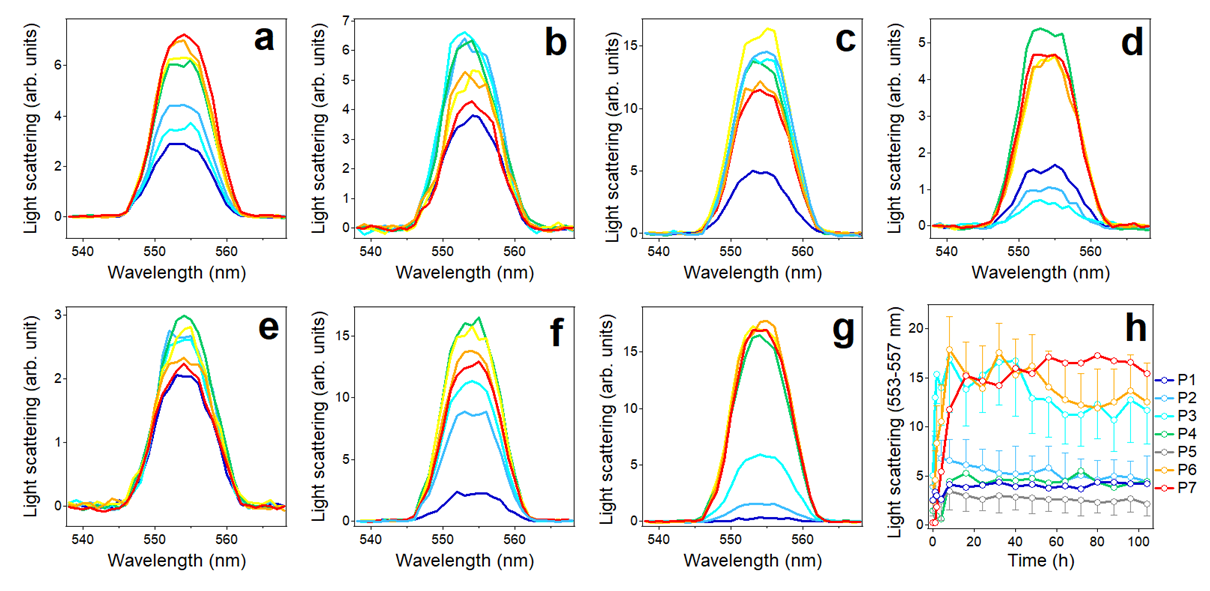


**Figure S3.** Time progression of light scattering spectra of peptides P1 (a), P2 (b), P3 (c), P4 (d), P5 (e), P6 (f), and P7 (g) at 70 μM. Color code in panels (a) through (g) is the same as in Figure 5. Panel (h) shows time course of light scattering for all seven peptides. Standard deviation bars from three experiments are only shown for selected peptides to maintain clarity. Error bars for other peptides are of similar magnitude.


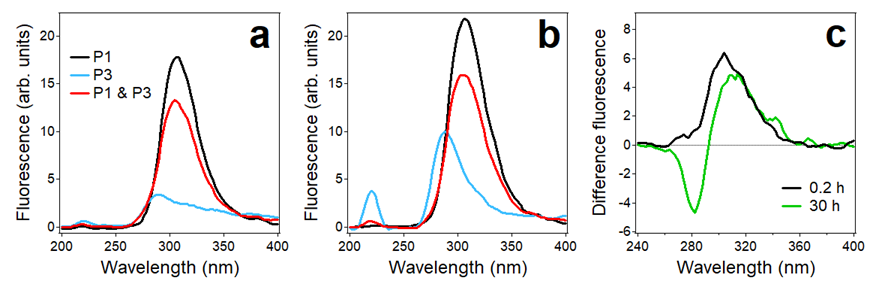


**Figure S4.** (a) and (b): Fluorescence spectra of P1 and P3 separately (each at 100 μM) and in combination (each at 50 μM) incubated in aqueous buffer (25 mM NaCl , 20 μM ThT, 25 mM Na,K-phosphate, pH 7.2) for 0.2 h (a) and 30 h (b). (c): Spectra of P1 and P3 were subtracted from the spectrum of the combination multiplied by 2, at 0.2 h and 30 h of incubation, as indicated. Spectra have been smoothed by 13-point Savitzky-Golay least-squares polynomial algorithm.


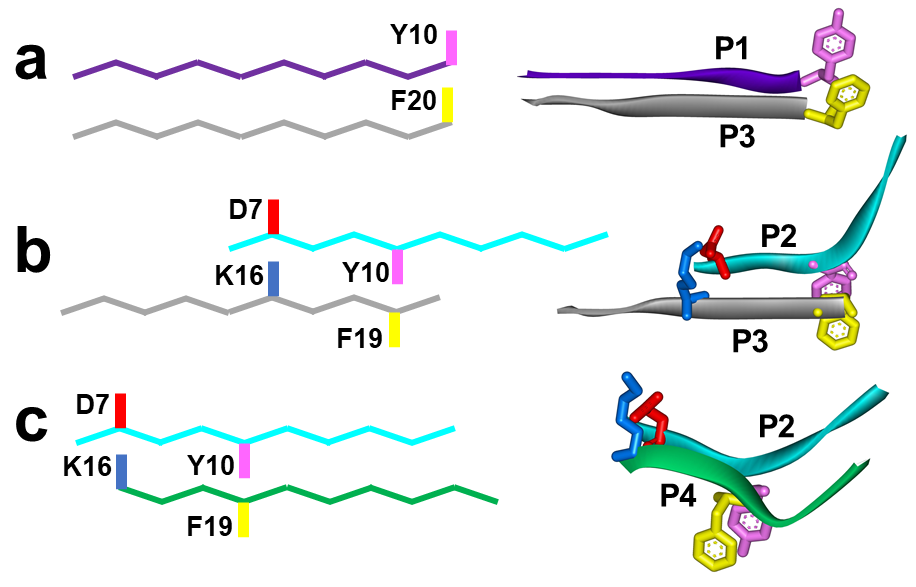


**Figure S5.** Models for interactions between peptides P1 and P3 (a), P2 and P3 (b), and P2 and P4 (c) based on fluorescence resonance energy transfer data. Peptide backbone is presented in line format in the left, with Tyr (Y), Phe (F), Asp (D), Lys (K) residues presented as vertical bars, and as ribbon in the right, with these residues presented in stick format, colored magenta (Tyr), yellow (Phe), red (Asp), and blue (Lys). Other side chains are omitted for clarity. Backbones of P1, P2, P3, and P4 are colored purple, cyan, grey, and green, respectively.


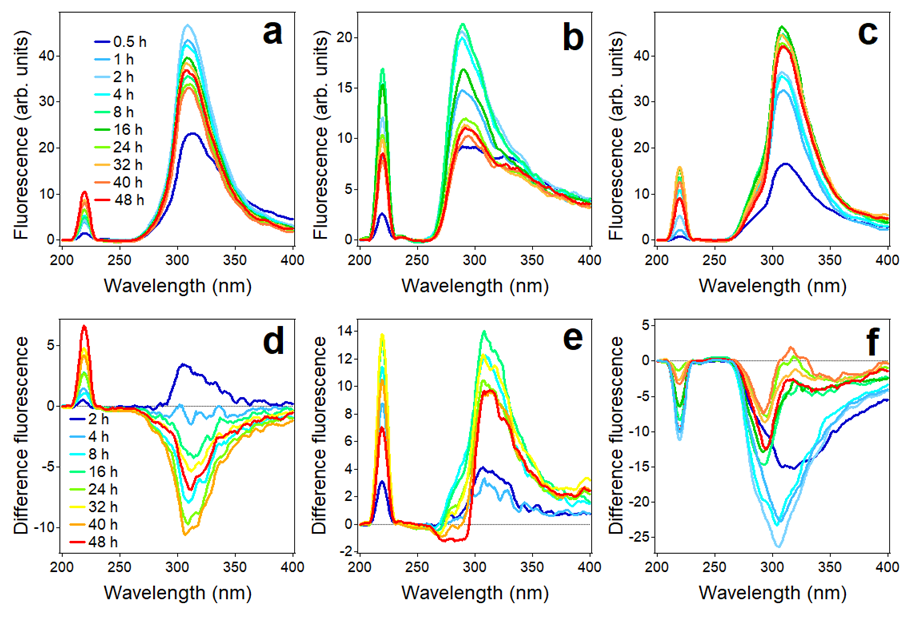


**Figure S6.** Fluorescence resonance energy transfer between P3 and Aβ_1-42_. Fluorescence spectra of 35 μM Aβ_1-42_ (a), 70 μM P3 (b), and 35 μM Aβ_1-42_ + 70 μM P3 (c) incubated in aqueous buffer (25 mM NaCl , 20 μM ThT, 25 mM Na,K-phosphate, pH 7.2) for time periods indicated in panel (a). Excitation was at 220 nm. (d): The spectrum of Aβ_1-42_ measured at 1 h subtracted from spectra of same peptide at indicated times. (e): The spectrum of combined sample (35 μM Aβ_1-42_ + 70 μM P3) at 1 h subtracted from spectra at later times, as indicated in panel (d). (f): Spectra of Aβ_1-42_ and P3 subtracted from spectra of the combined sample at all time points, as indicated in panel (a). Color code of panels (b), (c), and (f) is the same as in panel (a), and color code in (e) is the same as in (d). Spectra have been smoothed by 13-point Savitzky-Golay least-squares polynomial algorithm.


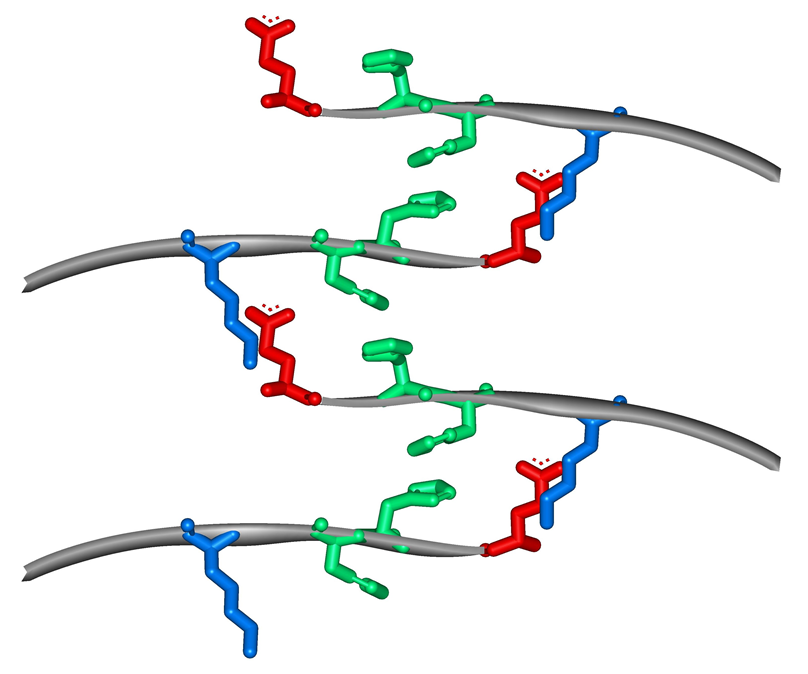


# Figure S7. Proposed mode of aggregation of P3. Red, blue, and green side chains are those of Glu11, Lys16, and His 13 and 14. All other side chains are omitted for clarity. The monomer β-strand structure is shown as a grey ribbon. The fibril would grow by means of intermolecular H-bonding perpendicular to the plane of the image.
